# Associations of Dietary-Lifestyle Patterns with Obesity and Metabolic Health: Two-Year Changes in MeDiSH^®^ Study Cohort

**DOI:** 10.3390/ijerph192013647

**Published:** 2022-10-21

**Authors:** Marta Lonnie, Lidia Wadolowska, Jakub Morze, Elzbieta Bandurska-Stankiewicz

**Affiliations:** 1 Department of Human Nutrition, Faculty of Food Science, University of Warmia and Mazury in Olsztyn, 10-718 Olsztyn, Poland; 2 Department of Internal Medicine, Faculty of Medical Sciences, University of Warmia and Mazury in Olsztyn, 10-561 Olsztyn, Poland

**Keywords:** adiposity, dietary-lifestyle patterns, men, metabolic, principal component analysis

## Abstract

This study aimed to evaluate changes in diet, adiposity, and metabolic outcomes after two years. In all, 358 Polish men aged 19–40 years old participated in the study. Data regarding dietary and lifestyle characteristics as well as family, socio-economic, and demographic status were collected using the food frequency questionnaire KomPAN^®^. Dietary lifestyle patterns were previously derived from data for 358 men by principal component analysis (PCA). Changes over time were examined in 95 men who returned after two years by calculating relative differences (RD, %) in mean values and markers distribution. Diet quality was described with two predefined scores: pro-Healthy-Diet-Index (pHDI) and non-Healthy-Diet-Index (nHDI). After two years, changes were observed in diet quality and metabolic health markers. No significant changes were observed in family, socio-economic, and demographic status, as well as other lifestyle factors. In the “sandwiches and convenience foods” pattern, an nHDI decrease (RD = −25.3%) was associated with a fasting blood glucose decrease (RD = −6.1%). In the “protein food, fried-food and recreational physical activity” and the “healthy diet, activity at work, former smoking” patterns, pHDI decreases (RD = −13.6% and −14.6%, respectively,) were associated with an adiposity increase. In the “fast foods and stimulants” pattern, no changes in pHDI and nHDI were observed, while adiposity markers and systolic blood pressure worsened. Conclusion: in the two-year perspective, dietary improvement was associated with improved glycemic control, despite no changes in body weight, while worsening of the diet quality or maintenance of unhealthy dietary behaviours were associated with the deterioration of metabolic health.

## 1. Introduction

Early adulthood begins in the early twenties and ends around the age of 40 [1]. During this critical stage of life, the biological, psychological, and societal transitions that occur (e.g., obtaining a degree, choosing a career, starting a family) can shape and establish long-term lifestyle behaviours, with a potentially detrimental effect on health [2,3]. It has been well documented that in this age group, the prevalence of some non-communicable diseases is higher in men, in particular, acute myocardial infarction, sudden cardiac death, stroke, and hypertensive diseases [4,5,6,7]. A large proportion of these cases may stem from an unhealthy lifestyle, which again, is more prevalent among young men [8]. In the American cohort of young adults (age 24–32), a significantly higher proportion of men than women presented risky health behaviours such as more frequent fast-food consumption, binge drinking, smoking, cannabis use, avoiding medical appointments, and illegal drug use [8]. These behaviours can have a lasting effect on health, especially if two or more behaviours coexist [9].

Historically, the effect of each lifestyle factor on health was investigated in isolation, in particular the effects of the so called ‘SNAP’–smoking, nutrition, alcohol, and physical activity [10]. In reality, people engage in a mixture of unhealthy or protective behaviours that may have additive or synergistic effects on health. Understanding the interactions between various (often unexpectedly co-occurring) behaviours might be crucial in identifying groups at risk and estimating the overall health risks. For example, a recent study showed that in older adults, prolong sitting times alone had little effect on all-cause mortality (HR = 1.15), while prolonged sitting among physically inactive adults increased this risk over twofold (HR = 2.42) [11]. It is not fully understood what the mechanistic background of lifestyle factor interactions are, but some have suggested that it could be due to a chronic inflammatory response triggered by joint exposure [12,13].

Fairly new statistical approaches in lifestyle science, based on exploratory data, allow for the capture of real-life-scenario clusters of behaviours in various populations, without pre-defined assumptions of which behaviours are expected to be coexistent [14]. This holistic approach of looking at lifestyle risk factors as clusters of behaviours has been previously used in relation to cardiometabolic health and obesity [15,16,17]; however, the clustering of lifestyle behaviours in young men have still not been fully explored.

It has been shown that dietary patterns are relatively stable over time. In the sample of American men aged 40–75, two major patterns were identified–Prudent and Western–which remained fairly stable when re-examined after a year (correlation coefficients of 0.70 and 0.67, respectively) [18]. The retention of unhealthy dietary behaviours may contribute to adverse health outcomes in later life. As it has been shown in the same cohort of men, after an eight-year follow-up, the risk of coronary heart disease displayed an increasing trend parallell to the increasing adherence to the Western pattern [19]. The studies on dietary pattern tracking (measuring consistency of dietary behaviours between at least two points in time, [20]) have also shown the worrying trend that diet quality worsens over time in adolescent males with adherence to the Western pattern as they enter adulthood, which is not as apparent among females of the same age [21].

The limitations of previous studies are twofold. The majority of the previous studies considered dietary or lifestyle behaviours in isolation [10,22,23]. In this study, we proposed the application of a holistic approach of looking at the empirically derived clusters of dietary patterns combined with lifestyle behaviours (physical activity at work and leisure time, smoking, alcohol use, and meal frequency) using an exploratory approach. This way, it is possible to reveal often unexpected combinations of behaviours, that truly exist in the studied population, and investigate how these unique clusters are associated with studied outcomes [24]. Secondly, dietary and lifestyle behaviours are sex- and age-specific [25]. While several studies examined dietary pattern tracking in adolescence or older age groups [26,27,28,29] not much research has been done with regard to males in early adulthood. Hence, we decided to focus on a group of men from a relatively narrow age group to provide a more focused view on this demographic group.

This study aimed to examine the changes in diet quality after two years’ time and the associations of those changes with adiposity and metabolic outcomes. Identifying groups at risk can help in the design of lifestyle interventions which target specific demographics and specific clusters of behaviours.

## 2. Materials and Methods

### 2.1. Study Sample

**Figure 1:**
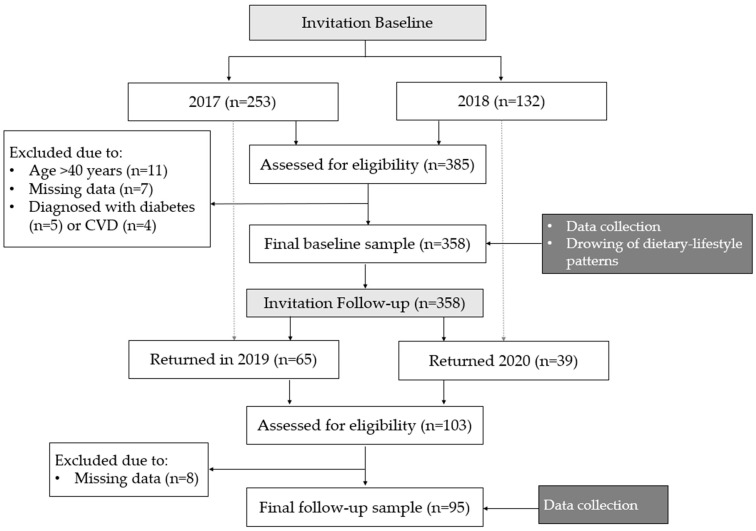
Study design and data collection.

A total of 358 men from the Warmia and Mazury region (Poland), aged 19–40 years old were analysed in this study (Figure 1) [30,31]. Details of the sample recruitment have been previously reported [31]. In brief, the study was publicly advertised using posters, social media advertising, and through direct contact with local businesses and council, inviting male employees to participate in the study. The main goal during recruitment was to obtain the maximum variability of the study sample in terms of sociodemographic characteristics to reflect the structure of the general population. The inclusion criteria were: males between 19 and 40 years old, with a cognitive ability to understand and respond to questions that were asked by the interviewer and who provided a written consent to participate. The exclusion criteria were: females, and a cognitive impairment that would prevent participants from understanding and responding to questions that were asked by the interviewer [30,31].

Data regarding adiposity and metabolic outcomes as well as family, socio-economic, and demographic status, and dietary and lifestyle characteristics were collected through structured interviews using the validated food frequency questionnaire KomPAN^®^ [32,33]. All data were collected in two time points during one-to-one interviews with trained researchers [31]. Baseline data were collected in 2017 (January to March) and 2018 (April to May), while follow-up data were collected in the Spring of 2019 and 2020.

### 2.2. Dietary and Lifestyle Behaviours

Dietary data examined the consumption frequency of foods commonly consumed in the Polish population, which were grouped into 25 food categories. The participants were asked to choose how often they consumed each type of food within the past 12 months. Available frequency answers were converted into daily frequencies and consisted of: never (0 times/day), 1–3 times a month (0.06 times/day), once a week (0.14 times/day), a few times a week (0.5 times/day), once a day (1.0 time/day), or a few times a day (2.0 times/day); more details can be found in the questionnaire manual guide [32].

Diet quality was described with two diet quality scores: pro-Healthy-Diet-Index (pHDI) and non-Healthy-Diet-Index (nHDI) [32,33]. Both diet quality scores were calculated as a sum of daily frequencies (in times/day) of food items consumption. The pHDI included 10 items representing potentially pro-healthy foods (wholemeal bread, wholegrain groats, milk, fermented milk drinks, cottage cheese, white meat, fish, legumes, fruit, vegetables) with the total score range: 0–20 times/day. The nHDI included 14 food items representing potentially unhealthy foods (white bread, refined groats, fast-foods, fried foods, butter, lard, cheese, cured meat, red meat, sweets, tinned meat, sweetened beverages, energy drinks, alcohol) with the total score range: 0–28 times/day. Both diet quality scores were converted to unify the total score range to 0–100 points for each of them. The following formulas were used [32,33]:

pHDI (in points) = (100/20) × the sum of frequency of 10 food items consumption (times/day)

nHDI (in points) = (100/28) × the sum of frequency of 14 food items consumption (times/day)

Lifestyle behaviours included daily meals frequency, level of physical activity, smoking, and screen time. The answer categories are displayed in Supplementary Material: Table S1.

### 2.3. Dietary-Lifestyle Patterns (DLPs)

**Figure 2:**
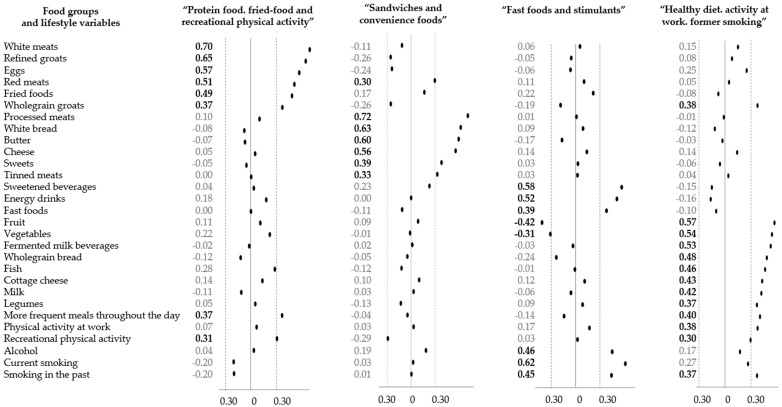
Black dots represent numerical values of factors loadings characterising each dietary-lifestyle pattern identified with principal component analysis [25]. Bold font represents items with factor loading ≥|0.30|.

The DLPs were previously derived from data for 358 men using Principal Component Analysis (PCA), with varimax normalized rotation [31,34]. A detailed description of identified DLPs was reported elsewhere [31]. In brief, 31 variables were included in the PCA consisting of 25 dietary variables and six lifestyle-related variables. To identify the final number of DLPs, the following criteria were considered: (i) the eigenvalues of at least 1.0, (ii) scree plot, and (iii) the total variance explained [34]. Items that had factor loadings ≥ |0.30| were used to label the patterns. The higher the values of factor loadings, the stronger association between dietary or lifestyle variables and the DLP. Four previously derived dietary-lifestyle patterns explained 33.2% of the variance and were labelled as follows: “protein food, fried-food and recreational physical activity” (12.5% of the explained variance), “sandwiches and convenience foods” (7.8%), “fast foods and stimulants” (6.4%), “healthy diet, activity at work, former smoking” (5.5.%) [31]. Components of each of the DLPs can found in Figure 2. Next, based on tertile distribution, participants were categorised into three categories (lower, middle, upper tertile) reflecting the adherence to the patterns: the higher the tertile, the higher the adherence to the pattern.

### 2.4. Adiposity and Metabolic Outcomes

Adiposity and metabolic outcomes were investigated in two time points: at baseline and after two years. Details were previously described [31]. In brief, to measure body weight and body size, the International Society for Advancement of Kinanthropometry (ISAK) International Standards for Anthropometric Assessment guidelines were followed [35]. The equipment used included: a portable stadiometer SECA 220 (height), electronic digital scale SECA 799 (weight), stretch-resistant tape SECA 201 (waist circumference), SECA medical Body Composition Analyzer (mBCA) 515 (body composition, visceral fat tissue and muscle mass). Adiposity was assessed using commonly used anthropometric indices: overweight (body mass index, BMI = 25–29.9 kg/m^2^), central obesity (waist-to-height ratio, WHtR ≥ 0.5), and general obesity (body fat ≥ 25%) [36,37,38]. The median values (Me) were applied to assess excessive visceral fat tissue (≥Me of fat tissue volume, i.e., 1.565 l) and increased skeletal muscle mass (≥Me of body mass, i.e., 37%) [31].

The metabolic outcomes included the concentration of fasting blood glucose (FBG), triglycerides (TG), and total cholesterol (TC) in capillary blood. All tests were performed in the morning in a fasting state. The measurements of systolic (SBP) and diastolic blood pressure (DBP) were taken using electronic monitor (Omron M3 Intellisense Automatic Blood Monitor, Omron Healthcare, Mannheim, Germany) and determined in line with the National Institute for Health and Care Excellence (NICE) procedures [39]. Metabolic abnormalities were based on the following cut-off points: FBG ≥ 100 mg/dL, TG ≥ 150 mg/dL, TC ≥ 200 mg/dL, or systolic or diastolic blood pressure ≥130 or ≥85 mmHg, respectively [40,41,42]

### 2.5. Family, Socio-Economic and Demographic Variables

The family, socio-economic, and demographic statuses, were reflected using objective and subjective measures. The considered variables included the place of residence, economic status, and education. Variables referring to family status included: being in a relationship and having children. A detailed description of KomPAN^®^ questionnaire categories [32] can be found in the Supplementary Material.

### 2.6. Statistical Analysis

Categorical variables were presented as percentages and continuous variables as means with standard deviations (SDs). Before the statistical analysis, the normality of all variables was verified using Kolmogorov–Smirnov and Shapiro–Wilk tests. To investigate the differences between groups (e.g., between total sample vs. sub-sample; between before vs. after 2 years), Pearson’s chi-squared test was used for categorical variables and *t*-test for continuous variables (paired *t*-test when appropriate). To investigate the differences in health outcomes after two years, relative differences were calculated:

(1) $$\mathrm{relative}\;\mathrm{difference}\;(\mathrm{RD},\;\%)=\frac{Final\;value-Initial\;value}{Initial\;value}\;\times \;100$$

For all tests *p*-value < 0.05 was considered as significant. Statistical analyses were carried out using STATISTICA software (version 10.0 PL; StatSoft Inc., Tulsa, OK, USA; StatSoft Polska, Kraków, Poland).

## 3. Results

### 3.1. Sociodemographic Sample Characteristics

The mean age of all study participants at baseline (n = 358) was 30.1 years old (Table 1). In the total sample, a larger proportion was from urban areas (64%), with comfortable or wealthy economic status (73%), with higher education (58%) and working physically (58%). In terms of family status, 65% declared being in a relationship and 37% of men had children. The subsample of men who returned to the study after two years (n = 95) was examined against the total sample (n = 358). The differences at baseline were related to age (30.1 vs. 31.8, total sample vs. subsample at baseline, respectively), place of residence (36% vs. 25% from villages and towns) education (42% vs. 28% with secondary or lower education) and screen time (44% vs. 28% with 6 h or more/day). No differences were identified within the subsample (n = 95) at baseline and follow-up, apart from an expected increase in age of approx. two years. To address attrition bias, characteristics of drop out cohort were compared with the characteristics of men who returned for the study (Supplementary material; Table S2). No differences between the groups were detected in terms of lifestyle behaviours (with exception to screen time) and adiposity characteristics. The only differences were related to lipid profile, place of residence and education: those who did not return had lower cholesterol levels, higher triglyceride concentrations and were more likely to be younger, with lower education and living in small towns and rural areas (Table S2).

Differences were identified between younger and older age group within the sample at baseline, in terms of place of residence, education, relationship status and having children (Supplementary material: Table S3). Younger (19–30 year) and older (31–40 year) age groups did not differ in terms of economic status and type of work.

### 3.2. Sample Characteristics: Dietary, Adiposity and Metabolic Outcomes

The subsample of men who returned for the follow-up assessment (n = 95) did not differ significantly at baseline from the total baseline sample (n = 358). The only difference was observed in terms of percentage of men with elevated total cholesterol (45% in the subsample vs. 34% in the total sample), but no difference was observed when mean values between the groups were compared (*p* = 0.081). After two years, an increase was observed in the mean values of WC (90.50 cm vs. 92.7 cm, before and after, respectively), WHtR (0.50 vs. 0.51), SBP (126.9 mmHg vs. 132.1 mmHg), and the percentage of men with excess visceral fat tissue (53% vs. 69%, before and after respectively).

### 3.3. Changes in Family Socio-Economic Status, Demographic Status and Lifestyle Factors after 2-Years across the DLP Patterns

Relative differences in family socio-economic status, demographic status, and lifestyle factors after two years across the DLP patterns are presented in Table 2. No significant differences were observed over two years in the upper tertiles of each dietary-lifestyle pattern.

### 3.4. Changes in Diet, Adiposity and Metabolic Outcomes after 2-Years across the DLP Patterns

Relative differences in diet quality, adiposity, and metabolic outcomes after two years across the DLP patterns are presented in Table 3. Among men with higher adherence to the ‘protein food, fried-food and recreational physical activity’ pattern, a decrease in diet quality was observed, expressed as lower scores of pHDI (RD = −13.6%, *p* = 0.011) as well as an increase of the proportion of men with an excess of visceral fat tissue (RD = 68.3%, *p* = 0.024). Among men with higher adherence to the ‘sandwiches and convenience foods’ pattern a decrease in nHDI was observed (RD = −25.3%, *p* < 0.001) suggesting a reduction in unhealthy dietary behaviours (i.e., diet quality improvement). Also, a reduction in the mean value of FBG was observed (RD = −6.1%, *p* = 0.014). Among men with higher adherence to the ‘fast foods and stimulants’ pattern the diet quality measured by the pHDI and nHDI did not change after two years. A significant increase was observed across the mean values of WC, WHtR and SBP (RD = 4.4%, *p* = 0.003; RD = 4.5%, *p* = 0.003 and RD = 5.1%, *p* = 0.047, respectively), as well as a higher proportion of men with central obesity, the excess of visceral fat tissue that elevates SBP or DBP (RD = 45.8%, *p* = 0.002, RD = 36.8%, *p* = 0.020 and RD = 27.9%, *p* = 0.011, respectively). Unexpectedly, a decrease in the percentage of men with elevated FBG was observed (RD = −76.5%, *p* = 0.004). Lastly, among men with higher adherence to the ‘healthy diet, activity at work, former smoking’ pattern, a decrease in diet quality (for pHDI) was observed (RD = −14.6%, *p* = 0.005) as well as an increase in mean values of WC, WHtR and visceral fat tissue (RD = 3%, *p* = 0.045; RD = 3.4%, *p* = 0.024 and RD = 72.4%, *p* = 0.026 respectively). Also, an increase was observed in the percentage of men with the excess of visceral fat tissue (RD = 82.5%, *p* = 0.009).

## 4. Discussion

Our study provides an insight into changes in diet quality after two years and the associations of those changes with adiposity and metabolic outcomes. After two years, positive or negative changes in diet quality were observed in three out of four DLPs. Considering that none of socio-economic and demographic status factors as well as other lifestyle factors (smoking, physical activity, screen-time) changed significantly over the two-year period (Table 1), it can be assumed that any changes observed within the health outcomes were mainly diet- and age-related. Diet improvement (nHDI decrease) was observed only in men from the “sandwiches and convenient foods” DLP and was associated with improved fasting blood glucose level. This suggests that even small dietary changes, manifested by a reduction in the frequency of unhealthy foods consumption, over a relatively short period of time (two years) may help in regulating glucose homeostasis. Furthermore, we did not observe worsening of the adiposity or other metabolic outcomes in this group, which implies a successful body weight maintenance over the course of two years. When compared with baseline results, the mean BMI of men with the highest adherence to this pattern remained stable (26.1 kg/m^2^, before and after) and there was no significant increase in the proportion of men with overweight or obesity (Table S4). These findings are very promising, as one of the primary strategies for diabetes remission is weight loss of >10% of body weight [43]. In our sample we observed an improvement in glycemic control without changes in body weight, suggesting that the diet composition itself may have a protective effect. This supports our previous findings that diet composition is an independent factor in increasing metabolic risk in young adults [44].

The worsening of the diet (pHDI decrease) was observed in men from two DLPs: ‘protein food, fried-food and recreational physical activity’ and ‘healthy diet, activity at work, former smoking’. In the first group, a decrease in diet quality was only associated with the increased proportion of men with an excess of visceral fat tissue, but this was not reflected in the mean value (before-after) comparison. Perhaps, the recreational physical activity diminished the impact of negative dietary changes on health. It needs to be noted that when the pHDI decreased, the nHDI did not increase, which can be interpreted as a slight reduction in the frequency of healthy food consumption (included in the pHDI score), but no increase in the consumption of unhealthy foods (included in the nHDI score). In the second group, the decrease in the pHDI was associated with the worsening of central obesity paameters (WC, WHtR and volume of visceral fat tissue). Men with the highest adherence to this pattern were characterised as physical workers, which may imply that there might be different responses to dietary changes, depending on the type of physical activity. This is an interesting finding which seemed to be supported by the results from a recent meta-analysis, which has shown that high levels of occupational physical activity may have a detrimental effect on men’s health, even when adjusted for confounding factors [45]. The authors found that high level of occupational physical activity increased the risk of early mortality in men by 18%, compared to those with lower levels of physical activity at work [45].

The diet quality of men from the ‘fast foods and stimulants’ remained stable; there were no changes in both scores, pHDI and nHDI. This pattern was interpreted as having potentially negative impacts on health, therefore its negative impact on health was expected. The current study demonstrated that the pattern was associated with an increase in central adiposity parameters after two years. Moreover, an increase was observed in the systolic blood pressure. Given this DLP composition, which included energy drinks, alcohol, fast foods (high in sodium) as well as current and past smoking, this is not an unexpected finding. All components of the pattern could be considered as stimulants with a documented hypertensive effect on blood pressure [46,47,48,49]. Worryingly, the adherence to this pattern was higher in younger men (18–30 years old), and yet, the changes have already started to manifest, after only two years, potentially triggering other pro-inflammatory processes which may resurface as they age, if the dietary behaviours remain unchanged.

### Strenghts and Limitations

The main strength of the study is a comprehensive approach in looking at lifestyle behaviours and their associations with metabolic health. Apart from the diet, the study took into account other lifestyle behaviours with documented effect on health (physical activity, smoking, alcohol and daily screen time) as well as family socio-economic and demographic factors. Since none of the above confounding factors, apart from age, seemed to change significantly over the two-year period, it allowed us to conclude that diet was an isolated factor potentially contributing to health-related changes.

The main limitation of our study is a relatively small sample size, however, there are some indicators that conclusions can be drawn with a reasonable amount of confidence. Firstly, dietary-lifestyle patterns were derived from the baseline sample of 358 men. Considering that the suggested rule of thumb regarding subject-to-item ratio in PCA analysis should be at least 10:1 [50], in our study, the ratio was 14:1. Secondly, just over a ¼ of men (95) from the baseline sample returned for the follow-up data collection after two years. We have performed several comparison analyses: (i) total sample vs. follow up sample at baseline; (ii) follow-up sample at baseline vs. follow-up sample after two years (Table 1); and (iii) follow-up sample at baseline vs. drop-out (Table S2). The latter analysis was key for identifying the differences between men who returned for the study and those who did not. No differences between the groups were detected in terms of lifestyle behaviours (with the exception of screen time) and adiposity characteristics, which we believe were the most crucial for the main analysis. The changes regarding screen time could be related to the fact that the follow-up recruitment that was carried out between the lockdowns, when people changed their screen-related routines. The only differences in metabolic health between drop out and those who returned were related to lipid profiles: those who did not return had lower cholesterol levels and higher triglyceride concentrations, which again seems inconsistent in terms of concluding that those with health concerns were more likely to return or leave. We do agree that attrition bias might be a concern due to differences in socioeconomic differences. Among those who did not return, a higher percentage was in a younger age group, lower education group and lived in small towns and rural areas. Despite potential concerns regarding the study’s internal and external validity, the results reflected physiological changes in young men based on their diet trajectory and described an alternative approach for studying the effects of lifestyle behaviours on health.

Thirdly, it might be argued that the number of participants in the follow-up cohort within each tertile of the dietary-lifestyle patterns was not prolific, ranging from 23 to 41 men. However, as explained in Section 2.6, the post hoc analysis proved these numbers to be sufficient for the analysis of changes in mean values but should be carefully interpreted in terms of changes in percentage distributions of the sample. Sample sizes of around 30 subjects can be sufficient to detect dietary changes if a reliable dietary intake assessment tool is used. In our study we used the validated KomPAN^®^ questionnaire [26] and its predefined diet quality scores, previously used in various populations [33,51]

Lastly, the study is of cross-sectional nature with added longitudinal perspective, focusing on young men, hence the results should not be widely generalised. On the other hand, it is a comprehensive snapshot of the complex matrix of diet and lifestyle behaviours interlinked with health and family outcomes in this specific demographic.

## 5. Conclusions

In the 2-year perspective, positive and negative changes were observed in diet quality. Improvement in diet quality was associated with improved glycemic control, despite no changes in body weight. Worsening of the diet quality or maintenance of unhealthy dietary behaviours were associated with deterioration of metabolic health. The key message from this study is that even small changes in diet over a short period of time can have an effect on adiposity and metabolic outcomes in young men. Hence, when designing lifestyle interventions, the evidence-based approach should be considered to best address the needs and specificity of the target group. Further studies are needed to understand the biological, social, and psychological reasons for the clustering of lifestyle behaviours in sex and-age specific groups as well as to provide insight into the mechanisms of the synergistic and cumulative effects on health.

## Figures and Tables

**Table 1:** Total sample and sub-sample characteristic: family socio-economic, demographic status lifestyle factors, diet, adiposity and metabolic outcomes in total sample and sub-sample (means and SD or number of subjects and % of the sample).

Variables	Total Sample	Sub-Sample before	*p*-Value ^#^	Sub-Sample after 2 Years	*p*-Value ^§^
Number of subjects	358	95		95	
Socio-economic and demographic status					
Age (years) ^¥^	30.1 (5.9)	31.8 (5.3)	0.011	33.7 (5.3)	0.014
*Age groups:* n (%)			0.001		0.035
19–30 years	43	25		13	
31–40 years	57	75		83	
*Place of residence*			0.044		0.626
Villages and towns	36	25		22	
Big cities	63	75		78	
*Economic status*			0.439		0.214
Modest	27	31		23	
Comfortable or wealthy	73	69		77	
*Education*			0.013		0.530
Secondary or lower	42	28		24	
Higher	58	72		76	
Family status (%)					
*In relationship*			0.141		0.524
Yes	65	73		77	
No	35	27		23	
*Having children*			0.285		0.129
Yes	37	43		54	
No	63	57		46	
Lifestyle factors					
*Number of meals per day*			0.268		0.529
Three or less	34	28		33	
Four or more	66	72		67	
*Physical activity at work or school*			0.862		0.783
Low	50	51		53	
Moderate or high	50	49		47	
*Rereational physical activity*			0.354		0.054
Low	16	20		10	
Moderate or high	84	80		90	
*Current smoking*			0.471		0.824
Yes	16	13		12	
No	84	87		88	
*Smoking in the past*			0.722		1.000
Yes	39	37		37	
No	61	63		63	
*Screen time*			0.015		1.000
6 h per day or more	44	58		58	
Less than 6 h	56	42		42	
Diet quality scores: mean (SD)					
pHDI	25.4 (11.6)	25.0 (10.8)	0.761	24.4 (9.8)	0.555
nHDI	19.7 (7.9)	18.9 (8.1)	0.371	17.4 (8.3)	0.086
Adiposity outcomes: mean (SD)					
BMI [kg/m^2^]	26.0 (3.7)	26.1 (3.2)	0.751	26.4 (3.6)	0.111
WC [cm]	89.9 (10.4)	90.5 (9.9)	0.642	92.7 (10.4)	0.002
WHtR [–]	0.50 (0.1)	0.50 (0.1)	1.00	0.51 (0.06)	0.002
Body fat [%]	22.2 (6.8)	23.2 (6.7)	0.202	23.6 (6.4)	0.246
Visceral fat tissue [l]	1.96 (2.2)	2.1 (3.2)	0.620	2.2 (1.5)	0.731
Skeletal muscle mass [%]	38.8 (3.2)	38.3 (3.2)	0.177	37.8 (3.8)	0.063
Metabolic outcomes: mean (SD)					
FBG [mg/dL]	85.0 (13.4)	85.5 (12.3)	0.743	83.6 (14.3)	0.288
TG [mg/dL]	143.1 (99.3)	125.4 (75.8)	0.107	136.0 (87.4)	0.378
TC [mg/dL]	185.6 (40.2)	193.5 (34.8)	0.081	197.6 (42.1)	0.309
SBP [mmHg]	126.1 (12.0)	126.9 (13.2)	0.572	132.1 (13.0)	0.001
DBP [mmHg]	77.4 (9.5)	77.9 (9.4)	0.648	79.0 (9.1)	0.160

^¥^ mean (standard deviation, SD); *p*-value of Pearson’s chi-squared test (for categorical variables) or *t*-test (for continuous variables); ^#^ vs. total sample; ^§^ vs. sub-sample before.

**Table 2:** Relative differences (RD, %) for means or % of the sample within sub-sample with higher adherence to each pattern in family socio-economic and demographic status, by dietary lifestyle patterns ^#^: before vs. 2 years after.

Variables	Sub-Sample (n = 95)		Protein Food, Fried-Food and Recreational Physical Activity (n = 32)		Sandwiches and Convenience Foods (n = 31)		Fast Foods and Stimulants (n = 23)		Healthy Diet, Activity at Work, Former Smoking (n = 30)	
	RD	*p*-Value	RD	*p*-Value	RD	*p*-Value	RD	*p*-Value	RD	*p*-Value
**Diet quality scores**										
pHDI	8.3	0.555	−13.6	0.011	18.6	0.576	14.8	0.953	−14.6	0.005
nHDI	1.3	0.086	−0.8	0.270	−25.3	<0.001	−7.0	0.151	6.1	0.328
**Socio-economic and demographic status**										
Age (years) ^¥^	6	0.014	6	0.187	6	0.142	6	0.306	6	0.119
Age 19–30 years (vs. 31–40 years)	−48	0.035	−29	0.396	−17	0.756	−23	0.536	−26	0.519
Place of residence: Villages and towns (vs. big cities)	−12	0.626	−11	0.777	−10	0.776	−37	0.326	35	0.519
Economic status: Modest (vs. comfortable or wealthy)	−26	0.214	0	1.00	−54	0.082	−57	0.153	0	1.00
Education: Secondary or lower (vs. higher)	−14	0.530	17	0.590	−34	0.374	−19	0.552	−15	0.766
**Family status**										
In relationship Yes (vs. No)	5	0.524	−48	0.140	−4	0.776	14	0.522	3	0.640
Having children Yes (vs. No)	26	0.129	20	0.453	33	0.200	46	0.238	8	0.780
**Lifestyle factors**										
Number of meals per day: 4 or more (vs. 3 or less)	18	0.529	433	0.086	−9	0.788	−19	0.552	143	0.228
Physical activity at work or school Low (vs. moderate or high)	4	0.783	32	0.434	7	0.793	23	0.552	21	0.592
Rereational physical activity Low (vs. moderate or high)	−50	0.054	0	1.00	−54	0.082	−43	0.300	−100	0.313
Current smoking Yes (vs. No)	−8	0.824	117	0.391	−32	0.490	−49	0.116	0	1.00
Smoking in the past Yes (vs. No)	0	1.000	23	0.599	0	1.00	0	1.00	−9	0.602
Screen time 6 h per day or more (vs. less than 6 h)	0	1.000	21	0.606	0	1.00	−8	0.768	16	0.598

^#^ Comparison of the means or % of the sample within the higher levels of adherence (i.e., upper tertiles) to each dietary-lifestyle pattern; ^¥^ difference in mean (standard deviation, SD); *p*-value of Pearson’s chi-squared test (for categorical variables) or paired *t*-test (for continuous variables.

**Table 3:** Relative differences (RD, %) for means or % of the sample within sub-sample with higher adherence to each pattern in adiposity and metabolic outcomes by dietary lifestyle patterns ^#^: before vs. 2 years after.

Variables	Sub-Sample (n = 95)		Protein Food, Fried-Food and Recreational Physical Activity (n = 32)		Sandwiches and Convenience Foods (n = 31)		Fast Foods and Stimulants (n = 23)		Healthy Diet, Activity at Work, Former Smoking (n = 30)	
	RD	*p*-Value	RD	*p*-Value	RD	*p*-Value	RD	*p*-Value	RD	*p*-Value
**Diet quality scores**										
pHDI	8.3	0.555	−13.6	0.011	18.6	0.576	14.8	0.953	−14.6	0.005
nHDI	1.3	0.086	−0.8	0.270	−25.3	<0.001	−7.0	0.151	6.1	0.328
**Adiposity outcomes**										
BMI	1.1	0.111	1.6	0.251	0.4	0.903	2.4	0.076	1.1	0.222
WC	2.7	0.002	2.6	0.098	1.2	0.559	4.4	0.003	3.0	0.045
WHtR	2.6	0.002	2.5	0.085	1.0	0.608	4.5	0.003	3.4	0.024
Body fat	5.2	0.246	9.7	0.330	0.0	0.516	1.8	0.557	3.7	0.500
Visceral fat tissue	68.2	0.731	100.1	0.099	35.5	0.494	48.8	0.659	72.4	0.026
Skeletal muscle mass	−1.3	0.063	−1.2	0.248	−0.1	0.731	−1.0	0.280	−0.8	0.247
**Metabolic outcomes**										
FBG	−0.6	0.289	6.5	0.352	−6.1	0.014	−3.5	0.200	4.9	0.600
TG	31.5	0.378	37.1	0.284	34.3	0.183	14.2	0.680	18.5	0.939
TC	4.1	0.309	2.6	0.683	2.8	0.762	6.1	0.181	−0.6	0.626
SBP	3.4	0.001	3.1	0.088	3.4	0.062	5.1	0.047	3.0	0.058
DBP	1.9	0.160	1.8	0.301	−0.4	0.710	3.8	0.187	1.5	0.500
**Adiposity abnormalities occurence**										
Normal weight (BMI = 18.5–24.9 kg/m^2^)	−2.6	0.887	−10.7	0.207	−13.5	0.854	−23.1	0.674	0.0	0.578
Overweight (BMI = 25–29.9 kg/m^2^)	−3.8	0.783	−8.3		21.9		33.3		−8.2	
Obesity (BMI ≥ 30 kg/m^2^)	44.4	0.378	-		0.0		−22.7		233.3	
Central obesity (WC ≥ 102 cm)	5.9	0.853	0.0	1.00	−17.4	0.755	34.6	0.178	−57.1	0.554
Central obesity (WHtR ≥ 0.5)	23.3	0.168	29.3	0.316	0.0	1.00	45.8	0.002	54.1	0.121
General obesity (Body fat ≥ 25%)	2.6	0.887	54.5	0.266	−18.8	0.44	0.0	1.00	35.0	0.541
Excess of visceral fat tissue (≥Me, i.e., 1.565 l)	30.2	0.024	68.3	0.024	29.1	0.189	36.8	0.020	82.5	0.009
Increased skeletal muscle mass (≥Me, i.e., 37%)	−1.6	0.887	−11.1	0.376	0.0	1.00	−6.6	0.575	−7.2	0.519
**Metabolic abnormalities occurence**										
Elevated FBG (≥100 mg/dL)	−22.2	0.601	200.0	0.302	−76.9	0.162	−76.5	0.004	0.0	1.00
Elevated TG (≥150 mg/dL)	29.2	0.329	0.0	1.00	73.7	0.246	50.0	0.056	17.4	0.766
Elevated TC (≥200 mg/dL)	6.7	0.663	39.5	0.209	10.9	0.607	0.0	1.00	−20.0	0.436
Elevated SBP (≥130 mmHg) or DBP (≥85 mmHg)	31.8	0.059	55.3	0.080	37.8	0.203	27.9	0.011	54.1	0.121

^#^ Comparison of the means or % of the sample within the higher levels of adherence (i.e., upper tertiles) to each dietary-lifestyle pattern; *p*-value of Pearson’s chi-squared test (for categorical variables) or paired *t*-test (for continuous variables).

## Data Availability

The data presented in this study are available on request from the corresponding author.

## Supplementary Materials

The following supporting information can be downloaded at: https://www.mdpi.com/article/10.3390/ijerph192013647/s1. Table S1. Answer categories of lifestyle behaviours variables–supplement; Table S2. Comparison of participating men who returned for the follow-up study (sub-sample before) vs. men who did not return for the follow-up stage (drop out) (means and SD or number of subjects and % of the sample). Table S3. Characteristics of the total sample by age groups at baseline (number of subjects and % of the sample); Table S4. Adherence to upper tertiles of dietary-lifestyle patterns by family socioeconomic and demographic status: prevalence ratios (95% Confidence Intervals); higher vs. lower adherence; * *p* < 0.05. Table S5. “Protein food, fried-food and recreational physical activity” pattern: diet quality scores, family socio-economic and demographic status, and adiposity and metabolic outcomes at baseline and after 2 years across the adherence to the pattern; Table S6. “Sandwiches and convenience foods” pattern: diet quality scores, family socio-economic and demographic status, adiposity and metabolic outcomes at baseline and after 2 years across the adherence to the pattern; Table S7. “Fast foods and stimulants” pattern: diet quality scores, family socio-economic and demographic status, adiposity and metabolic outcomes at baseline and after 2 years across the adherence to the pattern; Table S8. “Healthy diet, activity at work, former smoking” pattern: diet quality scores, family socio-economic and demographic status, and adiposity and metabolic outcomes at baseline and after 2 years across the adherence to the pattern.
