# Supplementary material for: Associations of Dietary-Lifestyle Patterns with Obesity and Metabolic Health: Two-Year Changes in MeDiSH® Study Cohort
Source: Int J Environ Res Public Health. 2022 Oct 21;19(20):13647. doi: 10.3390/ijerph192013647 (PMC9603699; doi:10.3390/ijerph192013647)
Supplement: Supplementary file 1 [file ijerph-19-13647-s001.zip › ijerph-1860383-supplementary.pdf]

Supplementary Material

Table S1. Answer categories of lifestyle behaviours variables – supplement

Lifestyle Behaviours	Answer Categories to Choose From:
Daily meal frequency	Five answers from one meal a day to five or more meals a day
Physical activity at work or school	Low—over 70% of time sedentary
	Moderate—about 50% of time sedentary and 50% active
	Higher—about 70% of time active or physical labour of high intensity
Physical activity at leisure time	Low—mostly sedentary, watching TV, reading newspapers/book, light house works, walking for 1–2 h a week
	Moderate—walking, cycling, exercise, gardening, or other light intensity physical activity for 2–3 h a week
	Higher—cycling, running, gardening, or other sport activities that require physical activity for more than 3 h a week
Past smoking	Yes/No
Current smoking	Yes/No
Screen time	Six categories: < 2 h/day, 2 to < 4 h/day, 4 to < 6 h/day, 6 to < 8 h/day, 8 to < 10 h/day, and ≥ 10 h/day

Table S2. Comparison of participating men who returned for the follow-up study (sub-sample before).
vs. men who did not return for the follow-up stage (drop out) (means and SD or number of subjects and % of the sample).

Variables	Total Sample	Sub-Sample before	Drop Out	p-Value #
Number of subjects	358	95	263	
**Socio-economic and demographic status**				
Age (years) ¥	30.1 (5.9)	31.8 (5.3)	29.3 (6.0)	<0.0001
Age groups: n (%)				0.00003
19-30 years	155(43)	24(25)	131(50)	
31-40 years	203(57)	71(75)	132(65)	
Place of residence: n(%)				0.009
Villages and towns	130(36)	24(25)	106(40)	
Big cities	228(63)	71(75)	157(60)	
Economic status: n(%)				0.37997
Modest	97(27)	29(31)	68(26)	
Comfortable or wealthy	261(73)	66(69)	195(74)	
Education: n(%)				0.00189
Secondary or lower	150(42)	27(28)	123(47)	
Higher	208(58)	68(72)	140(53)	
**Family status n(%)**				
In relationship				0.07178
Yes	233(65)	69(73)	164(62)	
No	125(35)	26(27)	99(38)	
Having children				0.17833
Yes	134(37)	41(43)	93(35)	

No	224(63)	54(57)	170(65)	
**Lifestyle factors: n(%)**				
Number of meals per day				0.268
Three or less	120(34)	29(30)	91(35)	
Four or more	238(66)	66(70)	172(65)	
Physical activity at work or school				0.90472
Low	179(50)	48(51)	131(50)	
Moderate or high	179(50)	47(49)	13250	
Rereational physical activity				0.14366
Low	55(16)	19(20)	36(14)	
Moderate or high	303(84)	76(80)	227(86)	
Current smoking				0.306
Yes	57(16)	12(13)	45(17)	
No	301(84)	83(87)	218(83)	
Smoking in the past				0.69030
Yes	138(39)	35(37)	103(39)	
No	220(61)	60(63)	160(61)	
Screen time				0.00203
6 hours per day or moe	159(44)	55(58)	104(40)	
Less than 6 hours	199(56)	40(42)	159(60)	
**Diet quality scores: mean (SD)**				
pHDI	25.4 (11.6)	25.0 (10.8)	25.5(11.9)	0.694031
nHDI	19.7 (7.9)	18.9 (8.1)	20.0 (7.8)	0.237645
**Adiposity outcomes: mean (SD)**				
BMI [kg/m^2]	26.0 (3.7)	26.1 (3.2)	25.9(3.8)	0.686002
WC [cm]	89.9 (10.4)	90.5 (9.9)	89.7(10.6)	0.546546
WHtR [-]	0.50 (0.1)	0.50 (0.1)	0.50(0.1)	0.593086
Body fat [%]	22.2 (6.8)	23.2 (6.7)	21.9(6.8)	0.109554
Visceral fat tissue [l]	1.96 (2.2)	2.1 (3.2)	1.9(1.7)	0.358942
Skeletal muscle mass [%]	38.8 (3.2)	38.3 (3.2)	39.0(3.2)	0.083995
**Metabolic outcomes: mean (SD)**				
FBG [mg/dL]	85.0 (13.4)	85.5 (12.3)	84.9(13.8)	0.696104
TG [mg/dL]	143.1 (99.3)	125.4 (75.8)	149.5(106.0)	0.042771
TC [mg/dL]	185.6 (40.2)	193.5 (34.8)	182.8(41.7)	0.026585
SBP [mmHg]	126.1 (12.0)	126.9 (13.2)	125.8(11.6)	0.475601
DBP [mmHg]	77.4 (9.5)	77.9 (9.4)	77.2(9.6)	0.507978

[#] p-value of Pearson's chi-squared test for categorical variables and t-test for continues variables.
[¥] mean (standard deviation, SD); SD–standard deviation;

Assessment of family socio-economic, demographic status and lifestyle factors

Question regarding place of residence consisted of four answers to choose from: villagetown < 20,000, town 20,000 to 100,000, city > 100.000 inhabitants. Level of education had three answer categories to choose from: lower secondary, upper secondary, higher. The financial situation was assessed using the following question: 'How would you describe your household's overall situation?'. Five answers were offered to choose from: (i) 'we do not have enough money for basic needs'—nobody reported such situation; (ii) 'we have to be very careful with our daily budget'; (iii) 'we have enough money for our daily needs, but we need to budget for bigger purchases'; (iv) 'we have enough money for our needs without particular budgeting'; and, (v) 'we can afford some luxury' [21,23]. Variables regarding family status considered: being in a relationship (Yes vs. No) and having children (Yes vs. No). Age as a part of demographic characteristics was provided as a continuous variable. The same procedure was applied to the men returning for the follow-up assessment.

Table S3. Characteristics of the total sample by age groups at baseline (number of subjects and % of the sample)

	Total sample	19-30 years	31-40 years	p-value
**Number of subjects**	358 (100.0)	155 (43.3)	203 (56.7)	
**Socio-economic and demographic status**				
Place of residence				<0.001
Rural	130 (36.3)	74 (20.7)	49 (13.7)	
Urban	228 (63.7)	81 (22.6)	154 (43.0)	
Economic status				0.489
Modest	96 (26.8)	41 (11.5)	56 (15.6)	
Comfortable or wealthy	261 (72.9)	114 (31.8)	147 (41.1)	
Education				<0.001
Secondary or lower	150 (41.9)	99 (27.6)	51 (14.3)	
Higher	208 (58.1)	56 (15.6)	152 (42.5)	
**Family status**				
Relationship status				<0.001
Not in a relationship	125 (34.9)	90 (25.1)	35 (9.8)	
In a relationship	233 (65.1)	65 (18.2)	168 (46.9)	
Having children				<0.001
No	224 (62.6)	151 (42.2)	73 (20.4)	
Yes	134 (37.4)	4 (1.1)	130 (36.3)	

p-value of Pearson's chi-squared test.

Table S4. Adherence to upper tertiles of dietary-lifestyle patterns by family socioeconomic and demographic status: prevalence ratios (95% Confidence Intervals); higher vs lower adherence; $p<0.05$

**Variables**	**Protein food. fried-food and recreational physical activity (n=32)**		**Sandwiches and convenience foods (n=31)**		**Fast foods and stimulants (n=23)**		**Healthy diet. activity at work. former smoking (n=30)**	
	PR (95% CI)	p-value	PR (95% CI)	p-value	PR (95% CI)	p-value	PR (95% CI)	p-value
**Socio-economic and demographic status**								
Age (years) ¥	0.93(0.83-1.04)	ns.	1.04(0.93-1.17)	ns.	0.95(0.85-1.06)	ns.	1.01 (0.90-1.14)	ns.
Age *19-30 years (vs. 31-40 years)*	1.02(0.91-1.14)	ns.	1.03(0.91-1.15)	ns.	1.03(0.91-1.15)	ns.	0.98(0.87-1.10)	ns.
Place of residence: *Villages and towns (vs. big cities)*	0.96(0.86-1.08)	ns.	1.00(0.89-1.13)	ns.	1.01(0.89-1.13)	ns.	0.95(0.84-1.07)	ns.
Economic status: *Modest (vs. comfortable or wealthy)*	0.99(0.88-1.11)	ns.	1.00(0.90-1.12)	ns.	1.03(0.91-1.16)	ns.	1.05(0.93-1.18)	ns.
Education: *Secondary or lower (vs. higher)*	1.02(0.91-1.14)	ns.	1.00(0.90-1.12)	ns.	1.04(0.93-1.15)	ns.	1.06(0.95-1.18)	ns.
**Family status**								
In relationship *Yes (vs. No)*	0.99(0.88-1.12)	ns.	1.05(0.93-1.20)	ns.	1.01(0.89-1.14)	ns.	1.07(0.94-1.21)	ns.
Having children *Yes (vs. No)*	1.05(0.91-1.21)	ns.	1.01(0.88-1.16)	ns.	1.05(0.91-1.22)	ns.	1.04(0.90-1.20)	ns.

¥ age in years as continuous variable

Table S5. "Protein food, fried-food and recreational physical activity" pattern: diet quality scores, family socio-economic and demographic status, and adiposity and metabolic outcomes at baseline and after 2 years across the adherence [1] to the pattern.

Variables	Lower adherence (n=36)			Moderate adherence (n=27)			Higher adherence (n=32)		
	Baseline	After 2 yrs	p-value	Baseline	After 2 yrs	p-value	Baseline	After 2 yrs	p-value
**Diet quality scores: mean (SD)**									
pHDI	19.7(9.4)	21.6(9.1)	0.120	22.31(8.8)	24.5(8.2)	0.247	33.1(9.0)	27.6(11.1)	0.011
nHDI	18.3(8.1)	17.7(8.8)	0.601	19.3(7.8)	17.0(8.8)	0.204	19.1(8.6)	17.3(7.5)	0.270
**Socio-economic and demographic status: number of subjects (% of the sample)**									
Age (years) ¥	32.6(5.3)	34.5(5.4)	0.141	32.8(4.9)	35.0(5.4)	0.127	30.8(5.5)	32.7(5.6)	0.187
Age *19-30 years (vs. 31-40 years)*	8(22)	8(22)	1.00	6(22)	3(11)	0.273	10(31)	7(22)	0.396
Place of residence: *Villages and towns (vs. big cities)*	10(28)	9(25)	0.789	5(19)	4(15)	0.715	9(28)	8(25)	0.777
Economic status: *Modest (vs. comfortable or wealthy)*	12(33)	4(11)	0.023	6(22)	7(26)	0.750	11(34)	11(34)	1.00
Education: *Secondary or lower (vs. higher)*	13(36)	11(31)	0.617	3(11)	3(11)	1.00	11(24)	9(28)	0.590
**Family status: % of the sample (SD)**									
In relationship *Yes (vs. No)*	26(72)	24(67)	0.609	21(78)	22(81)	0.735	10(31)	5(16)	0.140
Having children *Yes (vs. No)*	14(39)	18(50)	0.343	13(48)	16(59)	0.413	14(44)	17(53)	0.453
**Lifestyle factors: number of subjects (% of the sample)**									
Number of meals per day: *3 or less (vs. 4 or more)*	23(64)	19(53)	0.339	3(11)	7(26)	0.161	1(3)	5(16)	0.086
Physical activity at work or school *Low (vs. moderate or high)*	23(64)	22(61)	0.808	15(56)	15(56)	1.00	10(31)	13(41)	0.434
Recreational physical activity *Low (vs. moderate or high)*	14(39)	7(19)	0.070	4(15)	1(4)	0.159	1(3)	1(3)	1.00
Current smoking *Yes (vs. No)*	7(19)	5(14)	0.527	3(11)	2(7)	0.639	2(6)	4(13)	0.391
Smoking in the past *Yes (vs. No)*	19(53)	15(42)	0.345	6(22)	8(30)	0.535	10(31)	12(38)	0.599
Screen time *6 hours per day or more (vs. less than 6 hours)*	23(64)	23(64)	1.00	21(78)	19(70)	0.535	11(34)	13(41)	0.606
**Adiposity outcomes: mean (SD)**									
BMI [kg/m²]	26.6(4.0)	26.9(4.4)	0.176	25.7(3.0)	25.7(3.2)	0.959	25.9(2.1)	26.3(2.6)	0.251
WC [cm]	91.9(11.6)	95.0(12.0)	0.005	88.9(9.6)	90.1(10.0)	0.386	90.1(8.0)	92.2(8.2)	0.098
WHtR [-]	0.51(0.06)	0.53(0.07)	0.009	0.49(0.05)	0.50(0.05)	0.340	0.49(0.03)	0.50(0.04)	0.085
Body fat [%]	25.01(6.9)	25.3(6.8)	0.749	22.9(6.8)	23.3(6.1)	0.537	21.2(6.1)	22.1(5.8)	0.330
Visceral fat tissue [l]	2.9(4.9)	2.6(1.8)	0.716	1.7(0.8)	2.1(1.2)	0.064	1.7(1.1)	2.0(1.1)	0.099
Skeletal muscle mass [%]	37.2(3.2)	36.4(4.7)	0.243	38.5(3.1)	38.2(2.8)	0.294	39.6(3.0)	39.0(2.9)	0.248
**Metabolic outcomes: mean (SD)**									
FBG [mg/dL]	88.0(13.2)	81.3(13.5)	0.013	86.0(12.0)	84.3(13.7)	0.532	82.2(11.2)	85.6(15.6)	0.352

TG [mg/dL]	145.1(105.6)	150.5(82.5)	0.818	115.1(48.3)	119.9(76.3)	0.777	112.0(46.0)	133.3(100.7)	0.284
TC [mg/dL]	190.8(30.9)	194.8(45.2)	0.209	200.5(42.1)	202.5(36.4)	0.808	190.5(32.4)	194.0(52.8)	0.683
SBP [mmHg]	128.7(14.7)	134.5(14.5)	0.038	125.2(11.8)	130.3(11.9)	0.044	126.2(12.6)	131.0(12.0)	0.088
DBP [mmHg]	78.6(10.3)	80.5(11.2)	0.173	78.6(10.8)	78.4(8.1)	0.881	76.6(6.8)	77.7(7.2)	0.301
**Adiposity abnormalities: number of subjects (% of the sample)**									
Normal weight (BMI = 18.5-24.9 kg/m^2)	17(47)	14(39)		10(37)	13(48)		9(28)	8(25)	
Overweight (BMI = 25-29.9 kg/m^2)	11(31)	14(39)	0.722	16(59)	13(48)	0.704	23(72)	21(66)	0.207
Obesity (BMI >= 30 kg/m^2)	8(22)	8(22)		1(4)	1(4)		0(0)	3(9)	
Central obesity (WC ≥ 102 cm)	9(25)	9(25)	1.00	1(4)	3(11)	0.299	3(9)	3(9)	1.00
Central obesity (WHtR ≥ 0.5)	18(50)	23(64)	0.234	10(37)	10(37)	1.00	13(41)	17(53)	0.316
General obesity (Body fat ≥ 25%)	18(50)	18(50)	1.00	11(41)	8(30)	0.393	7(22)	11(34)	0.266
Excess of visceral fat tissue (≥ Me, i.e., 1.565 l)	20(56)	27(75)	0.083	17(63)	17(63)	1.00	13(41)	22(69)	0.024
Increased skeletal muscle mass (≥ Me, i.e., 37%)	18(50)	19(53)	0.814	16(59)	17(63)	0.780	26(81)	23(72)	0.376
**Metabolic abnormalities: number of subjects (% of the sample)**									
Elevated FBG (≥ 100 mg/dL)	5(14)	2(6)	0.233	3(11)	2(7)	0.639	1(3)	3(9)	0.302
Elevated TG (≥ 150 mg/dL)	10(28)	14(39)	0.317	5(19)	7(26)	0.513	8(25)	8(25)	1.00
Elevated TC (≥ 200 mg/dL)	17(47)	15(42)	0.635	14(52)	14(52)	1.00	12(38)	17(53)	0.209
Elevated SBP (≥ 130 mmHg) or DBP (≥ 85 mmHg)	17(47)	22(61)	0.237	13(48)	14(52)	0.786	12(38)	19(59)	0.080

[1] Adherence to the dietary-lifestyle pattern is based on subjects' tertile distribution: bottom tertile = lower adherence (used as the reference), middle tertile = moderate adherence, upper tertile = higher adherence; [¥] mean (standard deviation, SD); p-value of Pearson's chi-squared test (for categorical variables) or paired T-test (for continuous variables).

Table S6. "Sandwiches and convenience foods" pattern: diet quality scores, family socio-economic and demographic status, adiposity and metabolic outcomes at baseline and after 2 years across the adherence [1] to the pattern.

Variables	Lower Adherence (n=34)			Moderate Adherence (n=30)			Higher Adherence (n=31)		
	Baseline	After 2 yrs	p-value	Baseline	After 2 yrs	p-value	Baseline	After 2 yrs	p-value
**Diet quality scores: mean (SD)**									
pHDI	28.1(11.6)	26.1(11.3)	0.291	24.4(9.2)	24.0(8.4)	0.802	22.0(10.7)	22.9(9.4)	0.576
nHDI	12.2(4.9)	13.4(7.4)	0.297	18.1(3.8)	19.2(7.0)	0.450	26.9(6.9)	20.0(9.0)	<0.001
**Socio-economic and demographic status: number of subjects (% of the sample)**									
Age (years) ¥	31.0(5.9)	32.9(6.0)	0.201	32.0(4.2)	33.9(4.3)	0.089	33.4(5.4)	35.5(5.8)	0.142
Age *19-30 years (vs. 31-40 years)*	12(35)	8(24)	0.287	7(23)	4(13)	0.317	7(23)	6(19)	0.756
Place of residence: *Villages and towns (vs. big cities)*	9(26)	10(29)	0.787	6(20)	3(10)	0.278	9(29)	8(26)	0.776
Economic status: *Modest (vs. comfortable or wealthy)*	11(32)	9(26)	0.594	7(23)	8(27)	0.766	11(35)	5(16)	0.082
Education: *Secondary or lower (vs. higher)*	11(16)	11(16)	1.00	7(23)	6(20)	0.754	9(29)	6(19)	0.374
**Family status: number of subjects (% of the sample)**									
In relationship *Yes (vs. No)*	23(68)	27(79)	0.271	23(76)	24(80)	0.754	23(74)	22(71)	0.776
Having children *Yes (vs. No)*	12(35)	14(41)	0.618	14(47)	17(57)	0.438	15(48)	20(64)	0.200
**Lifestyle factors: number of subjects (% of the sample)**									
Number of meals per day: *3 or less (vs. 4 or more)*	7(21)	14(41)	0.066	9(30)	7(23)	0.559	11(35)	10(32)	0.788
Physical activity at work or school *Low (vs. moderate or high)*	14(41)	15(44)	0.806	15(50)	15(50)	1.00	19(61)	20(65)	0.793
Recreational physical activity *Low (vs. moderate or high)*	3(9)	3(9)	1.00	5(17)	1(3)	0.086	11(35)	5(16)	0.082
Current smoking *Yes (vs. No)*	2(6)	3(9)	0.642	4(13)	4(13)	1.00	6(19)	4(13)	0.490
Smoking in the past *Yes (vs. No)*	10(29)	12(35)	0.604	9(30)	7(23)	0.559	16(52)	16(52)	1.00
Screen time *6 hours per day or more (vs. less than 6 hours)*	16(47)	17(50)	0.808	18(60)	17(57)	0.793	21(68)	21(68)	1.00
**Adiposity outcomes: mean (SD)**									
BMI [kg/m²]	26.1(2.3)	26.4(2.8)	0.301	26.1(3.5)	26.6(4.2)	0.048	26.1(3.7)	26.1(3.7)	0.903
WC [cm]	90.1(8.2)	92.2(7.7)	0.078	92.1(9.4)	94.0(11.8)	0.001	91.1(12.1)	91.9(11.6)	0.559
WHtR [-]	0.49(0.04)	0.50(0.04)	0.070	0.50(0.05)	0.52(0.07)	0.001	0.51(0.06)	0.51(0.06)	0.608
Body fat [%]	21.9(6.9)	23.0(6.2)	0.236	23.2(5.9)	24.0(6.4)	0.078	24.5(7.3)	24.0(6.6)	0.516
Visceral fat tissue [l]	1.6(1.0)	2.0(1.0)	0.044	1.9(1.2)	2.4(1.8)	0.006	2.9(5.3)	2.3(1.5)	0.494
Skeletal muscle mass [%]	39.1(3.3)	38.5(3.1)	0.196	38.3(2.8)	37.4(5.1)	0.171	37.6(3.3)	37.4(3.0)	0.731
**Metabolic outcomes: mean (SD)**									
FBG [mg/dL]	82.2(11.5)	83.4(17.6)	0.724	85.4(13.7)	84.6(13.5)	0.799	89.2(11.2)	82.9(10.9)	0.014
TG [mg/dL]	127.6(80.8)	129.2(105.2)	0.947	131.5(93.3)	140.5(75.9)	0.674	117.2(47.8)	139.2(78.2)	0.183

TC [mg/dL]	188.0(39.4)	188.1(53.0)	0.997	192.9(32.0)	203.8(29.9)	0.566	200.0(31.9)	202.2(38.0)	0.762
SBP [mmHg]	127.7(11.7)	129.8(10.3)	0.422	124.2(13.6)	133.1(15.5)	0.001	128.5(14.3)	133.7(12.9)	0.062
DBP [mmHg]	75.7(10.6)	78.1(9.7)	0.096	77.7(8.7)	78.7(8.5)	0.442	80.5(8.1)	80.1(9.3)	0.710
**Adiposity abnormalities: number of subjects (% of the sample)**									
Normal weight (BMI = 18.5-24.9 kg/m^2)	11(32)	12(35)		9(30)	9(30)		16(52)	14(45)	
Overweight (BMI = 25-29.9 kg/m2)	21(62)	17(50)	0.417	19(63)	19(63)	1.000	10(32)	12(39)	0.854
Obesity (BMI >= 30 kg/m2)	2(6)	5(15)		2(7)	2(7)		5(16)	5(16)	
Central obesity (WC ≥ 102 cm)	4(12)	3(9)	0.699	2(7)	6(20)	0.129	7(23)	6(19)	0.755
Central obesity (WHtR ≥ 0.5)	14(41)	17(50)	0.465	11(37)	17(57)	0.121	16(52)	16(52)	1.00
General obesity (Body fat ≥ 25%)	12(35)	13(38)	0.801	9(30)	12(40)	0.417	15(48)	12(39)	0.44
Excess of visceral fat tissue (≥ Me, i.e., 1.565 l)	17(50)	23(68)	0.139	16(53)	21(70)	0.184	17(55)	22(71)	0.189
Increased skeletal muscle mass (≥ Me, i.e., 37%)	24(71)	23(68)	0.793	20(67)	20(67)	1.00	16(52)	16(52)	1.00
**Metabolic abnormalities: number of subjects (% of the sample)**									
Elevated FBG (≥ 100 mg/dL)	1(3)	4(12)	0.163	4(13)	2(7)	0.389	4(13)	1(3)	0.162
Elevated TG (≥ 150 mg/dL)	9(26)	7(21)	0.567	8(27)	12(40)	0.273	6(19)	10(33)	0.246
Elevated TC (≥ 200 mg/dL)	13(38)	14(41)	0.804	13(43)	13(43)	1.00	17(55)	19(61)	0.607
Elevated SBP (≥ 130 mmHg) or DBP (≥ 85 mmHg)	15(44)	18(53)	0.467	13(43)	18(60)	0.197	14(45)	19(62)	0.203

[1] Adherence to the dietary-lifestyle pattern is based on subjects' tertile distribution: bottom tertile = lower adherence (used as the reference), middle tertile = moderate adherence, upper tertile = higher adherence; [¥] mean (standard deviation, SD); p-value of Pearson's chi-squared test (for categorical variables) or paired T-test (for continuous variables).

Table S7. "Fast foods and stimulants" pattern: diet quality scores, family socio-economic and demographic status, adiposity and metabolic outcomes at baseline and after 2 years across the adherence [1] to the pattern.

Variables	Lower adherence (n=31)			Moderate adherence (n=41)			Higher adherence (n=23)		
	Baseline	After 2 yrs	p-value	Baseline	After 2 yrs	p-value	Baseline	After 2 yrs	p-value
**Diet quality scores: mean (SD)**									
pHDI	31.4(10.1)	28.6(10.2)	0.184	22.5(10.7)	23.2(9.7)	0.645	20.7(7.8)	20.8(7.6)	0.953
nHDI	17.1(7.2)	15.5(6.2)	0.169	17.8(7.8)	17.1(9.1)	0.634	23.3(8.6)	20.4(8.8)	0.151
**Socio-economic and demographic status: number of subjects (% of the sample)**									
Age (years) ¥	32.7(4.0)	34.6(4.1)	0.073	32.3(5.7)	34.3(6.0)	0.114	30.9(6.1)	32.8(6.3)	0.306
Age *19-30 years (vs. 31-40 years)*	4(13)	4(13)	1.00	11(27)	7(17)	0.286	9(39)	7(30)	0.536
Place of residence: *Villages and towns (vs. big cities)*	5(16)	6(19)	0.740	11(27)	10(24)	0.800	8(35)	5(22)	0.326
Economic status: *Modest (vs. comfortable or wealthy)*	11(35)	8(26)	0.409	11(27)	11(27)	1.00	7(30)	3(13)	0.153
Education: *Secondary or lower (vs. higher)*	6(19)	5(16)	0.740	10(24)	9(22)	0.793	11(48)	9(39)	0.552
**Family status: number of subjects (% of the sample)**									
In relationship *Yes (vs. No)*	26(84)	26(84)	1.00	28(68)	30(73)	0.627	15(65)	17(74)	0.522
Having children *Yes (vs. No)*	15(48)	18(58)	0.445	17(41)	20(49)	0.506	9(39)	13(57)	0.238
**Lifestyle factors: number of subjects (% of the sample)**									
Number of meals per day: *3 or less (vs. 4 or more)*	6(19)	7(23)	0.755	10(24)	15(37)	0.230	11(48)	9(39)	0.552
Physical activity at work or school *Low (vs. moderate or high)*	15(48)	14(45)	0.799	24(59)	25(61)	0.822	9(39)	11(48)	0.552
Recreational physical activity *Low (vs. moderate or high)*	3(10)	1(3)	0.301	9(22)	4(10)	0.131	7(30)	4(17)	0.300
Current smoking *Yes (vs. No)*	0(0)	3(10)	0.076	2(5)	3(7)	0.644	10(43)	5(22)	0.116
Smoking in the past *Yes (vs. No)*	6(19)	7(23)	0.756	13(32)	12(29)	0.810	16(70)	16(70)	1.00
Screen time *6 hours per day or more (vs. less than 6 hours)*	17(55)	18(58)	0.798	26(63)	26(63)	1.00	12(52)	11(48)	0.768
**Adiposity outcomes: mean (SD)**									
BMI [kg/m²]	25.8(3.5)	25.9(4.4)	0.754	26.0(3.0)	26.3(3.1)	0.408	26.7(3.0)	27.2(3.0)	0.076
WC [cm]	88.7(9.6)	91.2(11.3)	0.073	90.9(9.9)	92.0(9.1)	0.284	92.0(10.3)	95.9(11.0)	0.003
WHtR [-]	0.49(0.06)	0.50(0.07)	0.063	0.50(0.05)	0.50(0.05)	0.327	0.51(0.05)	0.53(0.05)	0.003
Body fat [%]	21.7(6.2)	22.2(5.9)	0.360	23.3(6.9)	23.8(5.8)	0.529	25.0(7.0)	25.3(7.6)	0.557
Visceral fat tissue [l]	1.7(1.3)	2.0(1.7)	0.071	1.9(1.1)	2.2(1.1)	0.119	3.1(6.1)	2.6(1.5)	0.659
Skeletal muscle mass [%]	39.0(3.0)	37.9(5.0)	0.118	38.3(3.3)	38.1(2.8)	0.555	37.5(3.1)	37.2(3.6)	0.280
**Metabolic outcomes: mean (SD)**									
FBG [mg/dL]	82.7(12.0)	80.4(15.1)	0.541	85.0(12.0)	85.0(14.8)	0.991	90.2(12.5)	85.5(11.8)	0.120
TG [mg/dL]	125.8(95.2)	128.4(80.7)	0.904	121.1(74.1)	140.1(102.9)	0.368	132.7(45.6)	138.9(66.4)	0.680
TC [mg/dL]	189.1(23.1)	193.0(47.7)	0.699	194.7(39.2)	195.7(37.5)	0.857	196.1(23.1)	207.3(42.3)	0.181

SBP [mmHg]	127.3(12.0)	129.3(14.2)	0.397	124.2(12.0)	130.4(10.2)	0.007	131.0(15.9)	139.0(13.8)	0.047
DBP [mmHg]	79.8(8.4)	80.1(9.0)	0.795	75.8(8.7)	76.5(6.6)	0.474	79.2(11.2)	81.8(12.1)	0.187
**Adiposity abnormalities: number of subjects (% of the sample)**									
Normal weight (BMI = 18.5-24.9 kg/m^2)	13(42)	15(48)		14(34)	13(32)		9(39)	7(30)	
Overweight (BMI = 25-29.9 kg/m2)	16(52)	13(42)	0.721	25(61)	23(56)	0.496	9(39)	12(52)	0.674
Obesity (BMI >= 30 kg/m2)	2(6)	3(10)		2(5)	5(12)		5(22)	4(17)	
Central obesity (WC ≥ 102 cm)	3(10)	2(6)	0.641	4(10)	5(12)	0.724	6(26)	8(35)	0.178
Central obesity (WHtR ≥ 0.5)	9(29)	13(42)	0.288	21(51)	21(51)	1.00	11(48)	16(70)	0.002
General obesity (Body fat ≥ 25%)	8(26)	8(26)	1.00	18(44)	19(46)	0.824	10(43)	10(43)	1.00
Excess of visceral fat tissue (≥ Me, i.e., 1.565 l)	11(35)	18(58)	0.075	26(63)	30(73)	0.342	13(57)	18(78)	0.020
Increased skeletal muscle mass (≥ Me, i.e., 37%)	23(74)	23(74)	1.00	23(56)	23(56)	1.00	14(61)	13(57)	0.575
**Metabolic abnormalities: number of subjects (% of the sample)**									
Elevated FBG (≥ 100 mg/dL)	2(6)	2(6)	1.00	3(7)	4(10)	0.693	4(17)	1(4)	0.004
Elevated TG (≥ 150 mg/dL)	9(29)	7(23)	0.562	9(20)	13(32)	0.206	6(26)	9(39)	0.056
Elevated TC (≥ 200 mg/dL)	12(39)	13(42)	0.796	18(44)	20(49)	0.658	13(57)	13(57)	1.00
Elevated SBP (≥ 130 mmHg) or DBP (≥ 85 mmHg)	14(45)	15(48)	0.780	14(34)	22(54)	0.075	14(61)	18(78)	0.011

[1] Adherence to the dietary-lifestyle pattern is based on subjects' tertile distribution: bottom tertile = lower adherence (used as the reference), middle tertile = moderate adherence, upper tertile = higher adherence; [¥] mean (standard deviation, SD); p-value of Pearson's chi-squared test (for categorical variables) or paired T-test (for continuous variables).

Table S8. "Healthy diet, activity at work, former smoking" pattern: diet quality scores, family socio-economic and demographic status, and adiposity and metabolic outcomes at baseline and after 2 years across the adherence [1] to the pattern.

Variables	Lower Adherence (n=34)			Moderate Adherence (n=31)			Higher Adherence (n=30)		
	Baseline	After 2 yrs	p-value	Baseline	After 2 yrs	p-value	Baseline	After 2 yrs	p-value
**Diet quality scores: mean (SD)**									
pHDI	15.1(5.2)	18.8(7.8)	0.003	24.3(4.6)	24.6(7.9)	0.862	36.9(8.0)	30.5(10.1)	0.005
nHDI	19.7(7.9)	19.4(9.0)	0.787	19.8(8.3)	17.1(8.1)	0.110	16.9(8.1)	15.4(7.4)	0.328
**Socio-economic and demographic status: number of subjects (% of the sample)**									
Age (years) [¥]	31.2(5.6)	33.0(5.6)	0.185	32.1(5.5)	34.1(5.5)	0.156	33.1(4.8)	35.2(5.4)	0.119
Age *19-30 years (vs. 31-40 years)*	10(29)	8(24)	0.582	7(23)	5(16)	0.520	7(23)	5(17)	0.519
Place of residence: *Villages and towns (vs. big cities)*	10(29)	8(24)	0.582	9(29)	6(19)	0.374	5(17)	7(23)	0.519
Economic status: *Modest (vs. comfortable or wealthy)*	11(32)	7(21)	0.271	11(35)	8(26)	0.409	7(23)	7(23)	1.00
Education: *Secondary or lower (vs. higher)*	8(24)	7(21)	0.770	11(35)	9(29)	0.587	8(27)	7(23)	0.766
**Family status: number of subjects (% of the sample)**									
In relationship *Yes (vs. No)*	21(62)	21(62)	1.00	21(68)	24(77)	0.393	27(90)	28(93)	0.640
Having children *Yes (vs. No)*	11(32)	14(41)	0.451	14(45)	20(65)	0.126	16(53)	17(57)	0.780
**Lifestyle factors: number of subjects (% of the sample)**									
Number of meals per day: *3 or less (vs. 4 or more)*	17(50)	15(44)	0.627	8(26)	11(35)	0.409	2(7)	5(17)	0.228
Physical activity at work or school *Low (vs. moderate or high)*	25(74)	23(68)	0.594	13(42)	15(48)	0.610	10(33)	12(40)	0.592
Recreational physical activity *Low (vs. moderate or high)*	12(35)	6(18)	0.099	6(19)	3(10)	0.279	1(3)	0(0)	0.313
Current smoking *Yes (vs. No)*	1(3)	3(9)	0.303	7(23)	4(13)	0.319	4(13)	4(13)	1.00
Smoking in the past *Yes (vs. No)*	10(29)	8(24)	0.582	13(42)	13(42)	1.00	12(40)	14(47)	0.602
Screen time *6 hours per day or more (vs. less than 6 hours)*	26(76)	25(74)	0.779	18(58)	17(55)	0.798	11(37)	13(43)	0.598
**Adiposity outcomes: mean (SD)**									
BMI [kg/m^2]	25.5(3.0)	25.6(3.0)	0.541	26.5(3.9)	26.9(4.6)	0.320	26.4(2.5)	26.7(2.7)	0.222
WC [cm]	89.3(8.8)	90.8(9.5)	0.147	92.0(12.1)	94.8(13.6)	0.049	90.3(8.7)	92.6(6.8)	0.045
WHtR [-]	0.49(0.05)	0.50(0.05)	0.180	0.51(0.06)	0.52(0.08)	0.067	0.49(0.04)	0.51(0.03)	0.024
Body fat [%]	23.8(6.7)	23.5(6.2)	0.644	23.6(7.9)	25.0(7.4)	0.110	21.9(5.4)	22.4(5.2)	0.501
Visceral fat tissue [l]	1.9(1.1)	2.1(1.2)	0.125	3.0(5.3)	2.7(2.0)	0.727	1.6(0.8)	2.0(1.0)	0.026
Skeletal muscle mass [%]	37.7(3.1)	37.4(4.9)	0.570	38.1(3.7)	37.2(3.4)	0.033	39.3(2.5)	38.9(2.5)	0.247
**Metabolic outcomes: mean (SD)**									
FBG [mg/dL]	86.4(11.9)	81.2(14.4)	0.039	86.9(13.6)	85.0(13.9)	0.535	83.0(11.4)	84.9(14.5)	0.600
TG [mg/dL]	131.9(88.8)	132.8(72.7)	0.965	118.3(55.3)	151.4(108.5)	0.144	125.4(79.8)	123.9(78.6)	0.939
TC [mg/dL]	193.6(32.7)	197.8(35.6)	0.428	188.5(35.1)	201.0(40.8)	0.067	198.5(37.3)	194.0(50.7)	0.626

SBP [mmHg]	126.6(14.5)	133.5(13.0)	0.023	127.4(13.1)	131.8(13.9)	0.121	126.5(12.2)	130.9(12.2)	0.058
DBP [mmHg]	77.6(9.5)	79/1(8.5)	0.196	78.3(12.0)	79.0(12.5)	0.637	77.9(5.9)	78.7(5.1)	0.499
**Adiposity abnormalities: number of subjects (% of the sample)**									
Normal weight (BMI = 18.5-24.9 kg/m^2)	17(50)	18(53)		12(39)	10(32)		7(23)	7(23)	
Overweight (BMI = 25-29.9 kg/m2)	13(38)	13(38)	0.918	15(48)	15(48)	0.748	22(73)	20(67)	0.578
Obesity (BMI >= 30 kg/m2)	4(12)	3(9)		4(13)	6(19)		1(3)	3(10)	
Central obesity (WC ≥ 102 cm)	3(9)	5(15)	0.452	8(26)	9(29)	0.776	2(7)	1(3)	0.554
Central obesity (WHtR ≥ 0.5)	14(41)	16(47)	0.625	16(52)	17(55)	0.799	11(37)	17(57)	0.121
General obesity (Body fat ≥ 25%)	16(47)	13(38)	0.462	14(45)	16(52)	0.611	6(20)	8(27)	0.541
Excess of visceral fat tissue (≥ Me, i.e., 1.565 l)	20(59)	22(65)	0.618	18(58)	22(71)	0.289	12(40)	22(73)	0.009
Increased skeletal muscle mass (≥ Me, i.e., 37%)	18(53)	21(62)	0.462	17(55)	15(48)	0.611	25(83)	23(77)	0.519
**Metabolic abnormalities: number of subjects (% of the sample)**									
Elevated FBG (≥ 100 mg/dL)	2(6)	2(6)	1.00	5(16)	3(10)	0.449	2(7)	2(7)	1.00
Elevated TG (≥ 150 mg/dL)	7(21)	9(26)	0.567	9(29)	12(39)	0.421	7(23)	8(27)	0.766
Elevated TC (≥ 200 mg/dL)	15(44)	19(56)	0.332	13(42)	15(48)	0.610	15(50)	12(40)	0.436
Elevated SBP (≥ 130 mmHg) or DBP (≥ 85 mmHg)	15(44)	19(56)	0.332	16(52)	19(62)	0.442	11(37)	17(57)	0.121

[1] Adherence to the dietary-lifestyle pattern is based on subjects' tertile distribution: bottom tertile = lower adherence (used as the reference), middle tertile = moderate adherence, upper tertile = higher adherence; [¥] mean (standard deviation, SD); p-value of Pearson's chi-squared test (for categorical variables) or paired T-test (for continuous variables).
